# Correlation of tilt of the anterior pelvic plane angle with anatomical pelvic tilt and morphological configuration of the acetabulum in patients with developmental dysplasia of the hip: a cross-sectional study

**DOI:** 10.1186/s13018-019-1382-8

**Published:** 2019-10-17

**Authors:** Norio Imai, Hayato Suzuki, Asami Nozaki, Yuki Hirano, Naoto Endo

**Affiliations:** 1 Division of Comprehensive Geriatrics in Community, Graduate School of Medical and Dental Sciences, Niigata University, 1-757, Asahimachi-dori, Chuo Ward, Niigata City, Niigata Prefecture 9518510 Japan; 2 Division of Orthopedic Surgery, Department of Regenerative and Transplant Medicine, Graduate School of Medical and Dental Sciences, Niigata University, Niigata City, Japan

**Keywords:** Pelvic tilt, Developmental dysplasia of the hip, Cross-sectional study, Hip

## Abstract

**Background:**

It was previously reported that pelvises with developmental dysplasia of the hip are tilted anteriorly, which increases bony coverage of the femoral head. This study aimed to investigate the correlation between anatomical parameters of the pelvis such as pelvic incidence and anatomical pelvic tilt and functional parameters of the spine and pelvis such as tilt of the anterior pelvic plane.

**Methods:**

We examined 84 female patients with bilateral developmental dysplasia of the hip who had undergone curved periacetabular osteotomy at author’s institution. Radiographs of the thoracic to lumbar spines and the pelvis were obtained in the standing position to measure spino-pelvic parameters before surgery. Morphological parameters of the acetabulum such as the anterior center-edge (CE) angle, posterior CE angle, lateral CE angle, and acetabular anteversion were measured using a preoperative three-dimensional pelvic model reconstructed from computed tomography images. Pearson’s correlation analysis was conducted to evaluate the relationship of these parameters.

**Results:**

With regard to correlations between pelvic incidence (PI) and other parameters, the sacral slope (SS) value (*r* = 0.666) was the highest among functional parameters and the anatomical-SS value (*r* = 0.789) was the highest among morphological parameters. There were moderate correlations of the anterior pelvic plane angle (APPA) with pelvic tilt (PT) (*r* = − 0.594) and anatomical-PT (*r* = 0.646). With regard to correlations between spino-pelvic parameters and bony morphological parameters of the acetabulum, there was a moderate correlation between anatomical-PT and acetabular anteversion (AA) (*r* = 0.424). There were moderate correlations of APPA with the anterior CE angle (*r* = − 0.478), posterior CE angle (*r* = 0.432), and AA (*r* = 0.565). APPA had a stronger correlation with anatomical-PT (*r* = 0.646) than with AA.

**Conclusions:**

The tilt of the pelvis may be more dependent on anatomical-PT, a morphological parameter of the pelvis, than the lateral CE angle, anterior CE angle, posterior CE angle, and acetabular anteversion on bony coverage of the acetabulum. This study is the first to investigate the correlation between functional parameters of the pelvis and spine and morphological parameters of the pelvis and acetabulum besides PI.

## Background

Developmental dysplasia of the hip (DDH) is considered one of the more frequent causes of secondary osteoarthritis of the hip, especially in Japan [1]. A higher degree of tilt of the anterior pelvic plane angle (APPA) has been reported in patients with DDH than in normal subjects [2, 3], and it has been considered to increase bony coverage of the femoral head [4].

Pelvic incidence (PI) is independent of the spatial orientation of the pelvis, and it was considered to be a parameter that cannot be significantly changed according to age or sex [5]. Gebhart et al. [6] described that a higher PI value may affect the development of osteoarthritis of the hip. In contrast, Raphael et al. [7] reported that a higher PI value is not associated with osteoarthritis of the hip; thus, the relationship between PI and osteoarthritis of the hip is still unclear to date. Moreover, PI is well known to affect sagittal spinal balance such as lumbar lordosis (LL) and posture in standing position [8, 9].

In a previous study, Imai et al. [10] reported that PI and anatomical pelvic tilt (anatomical-PT), PT relative to APP, was larger in DDH patients than in normal healthy subjects in both groups by three-dimensional (3D) measurement. Moreover, they also described that there was a high correlation between PI and anatomical sacral slope (anatomical-SS) [10, 11]. However, the relationship between anatomical-PT, anatomical-SS, and APPA in that study is unclear.

Most of the intraoperative assistance systems of total hip arthroplasty (THA) or pelvis osteotomy, such as the computed tomography (CT)-based imageless navigation system [12, 13] or a mechanical support device [14], refer to the anterior pelvic plane (APP) or functional pelvic plane, which was the APPA in supine position [15]. If DDH patients tilted their pelvis anteriorly to increase the bony coverage of the femoral head [4], the degree of anterior tilt of the pelvis may be dependent on the anterior center-edge angle (ACE), posterior CE angle (PCE), lateral CE angle (LCE), and acetabular anteversion (AA) (operative anteversion of Murray’s definition [16]).

The purpose of this study was to investigate the correlation between anatomical parameters of the pelvis, PI, anatomical-SS, anatomical-PT, ACE, PCE, LCE, and AA, and functional parameters of the spine and pelvis, SS, pelvic tilt (PT), TK, LL, and APPA. The hypothesis of this study was that APPA would strongly correlate with the bony morphological parameters of the acetabulum such as ACE, PCE, LCE, and AA.

## Methods

This study was approved by the institutional research board of the university, and the need for informed consent was waived because this study was a retrospective cross-sectional study.

We examined 84 women with bilateral DDH from the author’s institution who had undergone curved periacetabular osteotomy [17] for the treatment of secondary osteoarthritis of the hip caused by DDH joint between April 1, 2008, and July 30, 2017. The CE angles of the hip joints of the included patients were less than 25°, as measured from the anteroposterior plain radiograph of the hip. We defined them as study participants because we speculated that these patients might have a typical morphological feature of DDH and typical functional alignment of the pelvis and spine. We excluded patients who had previously undergone hip surgery or those with Crowe stages 2–4 of subluxation or Tonnis grades 2–3 for arthritic changes identified from plain radiographs of the hip. Patients’ mean age was 35.0 ± 9.2 years (range 20–52 years), and the mean body mass index was 22.0 ± 2.9 kg/m^2^ (range 16.2–27.8 kg/m^2^).

CT scans of all participants were examined before curved periacetabular osteotomy to plan for osteotomy by reconstructing a 3D bone model. The radiographs of the thoracic to lumbar spines and pelvis in the standing position were also obtained before surgery to check for the existence of a vertebral anomaly and spinal sagittal alignment.

### Measurements of pelvic parameters

**Fig. 1:**
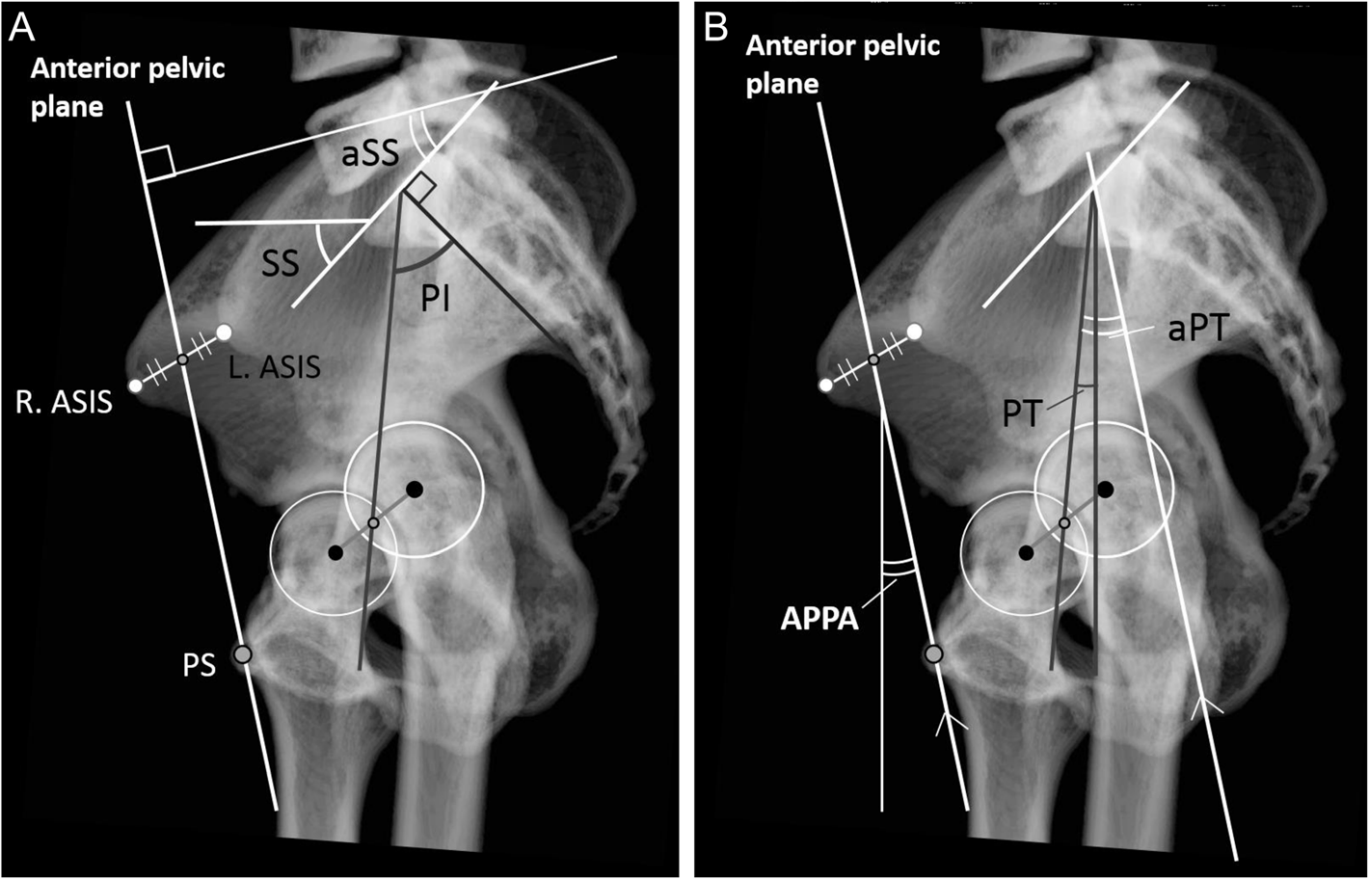
Measurement of pelvic parameters. Anatomical SS (**a**) and anatomical PT (**b**) were measured per the previous work of Imai et al. [14]. Pelvic parameters: PI, pelvic incidence; SS, sacral slope; APPA, anterior pelvic plane angle; L.ASIS, R.ASIS, left and right anterior superior iliac spine, respectively; PT, pelvic tilt

**Fig. 2:**
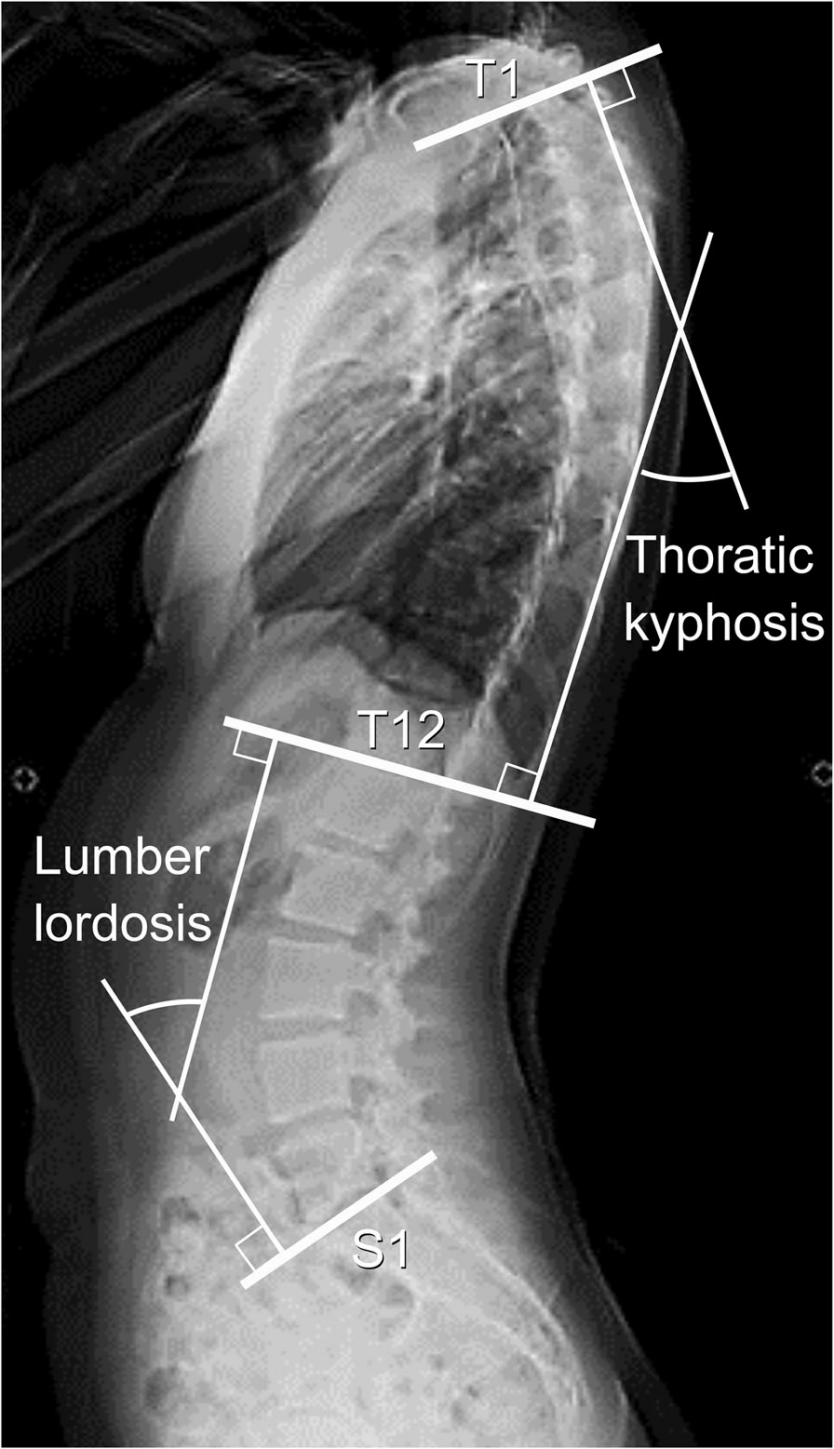
Measurements of spinal sagittal parameters. Lumber lordosis and thoracic kyphosis are the angles measured between the inferior endplate of T12 and superior endplate of S1 and the superior endplate of T1 and inferior endplate of T12, respectively

We measured PI, SS, PT, anatomical-SS, anatomical-PT, APPA, TK, and LL using standing thoracic and lumbar radiographs that included the pelvis on Picture Archiving and Communication Systems (PACS). First, with regard to the functional parameters, SS was defined as the angle between the superior endplate of S1 and the horizontal line projected in the sagittal plane (Fig. 1a). PT was defined as the angle between the line connecting the midpoint of the sacral plate to the hip axis and the vertical line projected in the sagittal plane (Fig. 1b). APPA was defined as the angle between the line connecting the midpoint of both anterior superior iliac spines (ASISs) to the pubic symphysis, which was the APP, and the vertical line of the lateral radiograph of the pelvis in the standing position [18] (Fig. 1b). LL was the angle measured between the inferior endplate of T12 and the superior endplate of S1. TK was the angle measured between the superior endplate of T1 and the inferior endplate of T12 (Fig. 2). Next, with regard to the anatomical parameters, PI was defined as the angle between the line perpendicular to the superior endplate of S1 and the line connecting the center of the endplate of S1 to the hip axis, which was the midpoint of the centers of both femoral heads, projected in the sagittal plane (Fig. 1a). Anatomical-SS was measured as the angle between the line connecting the line of the superior endplate of S1 and the line perpendicular to APP (Fig. 1a). Anatomical-PT was measured as the angle between the line parallel to APP from the midpoint of the superior endplate of S1 to the midpoint of both femoral head centers and APP (Fig. 1b).

**Fig. 3:**
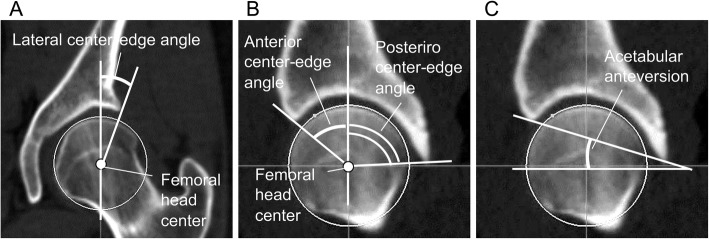
Measurement of morphological parameters of the acetabulum. Lateral center-edge angle (**a**), anterior and posterior center-edge angles (**b**), and acetabular anteversion (**c**) were measured in the coronal (**a**) and sagittal (**b**, **c**) planes through the femoral head center

ACE, PCE, LCE, and AA were measured using a 3D pelvic model reconstructed from CT images with ZedHip® software (Lexi, Tokyo, Japan). After adjusting the pelvic model to APP, LCE was defined as the angle between the vertical line and the line connecting the femoral head center of the lateral acetabular margin in the coronal plane [18] (Fig. 3a). ACE and PCE were defined as the angles between the vertical line and the line connecting the femoral head center and the anterior acetabular margin and the posterior margin in the sagittal plane, respectively [19] (Fig. 3b). AA was defined as the angle between the horizontal line and the line connecting the anterior and posterior acetabular margins in the sagittal plane [20] (Fig. 3c). ACE, PCE, LCE, and AA were measured bilaterally, and an average of the left and right values was expressed.

### Statistical analysis

We analyzed the data using SPSS statistical software (version 21; SPSS, Inc., Chicago, IL). Quantitative variables were computed as average ± standard deviation (range). Pearson’s coefficients were used to determine the correlation coefficients of pelvic parameters and spino-pelvic alignment such as PI, SS, PT, anatomical-SS, anatomical-PT, APPA, LL, TK, LCE, ACE, PCE, and AA according to Guilford’s definition: less than 0.2, no correlation; 0.2 to less than 0.4, weak correlation; 0.4 to less than 0.7, moderate correlation; and 0.7 and more, strong correlation [21].

We also analyzed the validity of the measurement values. Intraobserver reliability and interobserver reliability with intraclass correlation coefficients (ICCs) and two-sided 95% confidential intervals were calculated to evaluate validations. We measured values twice at more than 1-week intervals to determine intraobserver reliability. We compared the measurements performed by another observer with our measurements to assess the interobserver reliability. Statistical significance was considered as *p* < 0.01. We also performed a post hoc analysis to evaluate statistical power (type II (ß) error). We defined the effect size (*d*) as 0.3 and type I (*α*) error as 0.05 in the correlation analysis.

## Results

Details of the measured values are described in Table 1. APPA was 3.5 ± 9.3°, suggesting that the pelvis was 3.5° anteriorly tilted.

**Table 1:** Measurements of spino-pelvic and spinal parameters in 84 women with dysplasia of the hip

Parameter	Value
Functional	
APPA (°)	3.5 ± 9.3 (− 14.0–20.9)
SS (°)	43.8 ± 10.9 (16.1–69.0)
PT (°)	10.7 ± 9.0 (− 8.0–30.0)
TK (°)	35.0 ± 10.7 (7.0–67.0)
LL (°)	55.4 ± 18.4 (3.0–83.0)
Anatomical	
PI (°)	54.2 ± 10.6 (31.0–77.0)
Anatomical-SS (°)	40.6 ± 9.4 (20.0–61.0)
Anatomical-PT (°)	14.1 ± 9.4 (0.3–27.7)
LCE (°)	13.9 ± 5.9 (0.7–25.1)
ACE (°)	40.6 ± 9.5 (16.4–61.4)
PCE (°)	99.8 ± 18.4 (38.0–133.9)
AA (°)	29.6 ± 10.9 (− 4.9–50.3)

Data are presented as mean ± standard deviation (range)

*Anatomical-SS* anatomical sacral slope, *anatomical-PT* anatomical pelvic tilt, *LL* lumbar lordosis, *PI* pelvic incidence, *PT* pelvic tilt, *SS* sacral slope, *TK* thoracic kyphosis, *APPA* anterior pelvic plane angle, *LCE* lateral center-edge angle, *ACE* anterior center-edge angle, *PCE* posterior center-edge angle, *AA* acetabular anteversion

With regard to the correlations between PI and other parameters, the SS value (*r* = 0.666) was the highest in functional parameters, and the anatomical-SS value (*r* = 0.789, *p* < 0.01) was the highest in morphological parameters (Table 2). However, there was no strong correlation of PI with PT (*r* = 0.380) and anatomical-PT (*r* = 0.305) (Table 2).

**Table 2:** Correlations between pelvic parameters and sagittal spinal parameters

	Functional				Anatomical		
	SS	PT	TK	LL	PI	Anatomical-SS	Anatomical-PT
Functional							
APPA	0.317^*^	− 0.594^*^	0.207	0.340^*^	0.159	0.424^*^	0.646^*^
SS		− 0.323^*^	0.204	0.843^*^	0.666^*^	0.712^*^	− 0.062
PT	− 0.323^*^		0.217	0.328^*^	0.380^*^	0.251^*^	0.182
TK	0.204	0.217		0.463^*^	− 0.014	0.039	0.087
LL	0.843^*^	0.328^*^	0.463^*^		0.573^*^	0.598^*^	0.150
Anatomical							
PI	0.666^*^	0.380^*^	− 0.014	0.573^*^		0.789^*^	0.305^*^
Anatomical-SS	0.712^*^	0.251^*^	0.039	0.598^*^	0.789^*^		0.344^*^
Anatomical-PT	− 0.062	0.182	0.087	0.150	0.305^*^	0.344^*^	

*Anatomical-SS* anatomical sacral slope, *anatomical-PT* anatomical pelvic tilt, *LL* lumbar lordosis, *PI* pelvic incidence, *SS* sacral slope, *PT* pelvic tilt, *TK* thoracic kyphosis, *APPA* anterior pelvic plane angle

*p < 0.01

There were moderate correlations of APPA with PT (*r* = − 0.594) and anatomical-PT (*r* = 0.646). However, there was no strong correlation of APPA with PI (*r* = 0.159) and SS (*r* = 0.317) (Table 2).

There was a strong correlation of LL with SS (*r* = 0.843) and a moderate correlation of LL with anatomical-SS (*r* = 0.598) and TK (*r* = 0.463). However, there was no strong correlation between LL and APPA (*r* = 0.340) (Table 2).

With regard to the correlations between spino-pelvic parameters and bony morphological parameters of the acetabulum, there were moderate correlations between anatomical-PT and AA (*r* = 0.424) (Table 3). Moreover, there was a moderate correlation between APPA and LCE (*r* = − 0.360), ACE (*r* = − 0.478), PCE (*r* = 0.432), and AA (*r* = 0.565).

**Table 3:** Correlations between pelvic parameters and morphological parameters of the acetabulum

	ACE	PCE	LCE	AA
APPA	− 0.478^*^	0.432^*^	− 0.360^*^	0.565^*^
SS	− 0.133	0.220	− 0.187	0.275^*^
PT	0.241	− 0.224	− 0.004	− 0.283^*^
TK	− 0.235	0.060	0.128	0.163
LL	0.217	− 0.249	− 0.285^*^	0.293^*^
PI	0.070	0.045	− 0.347^*^	0.004
Anatomical-SS	0.235	− 0.053	− 0.398^*^	− 0.146
Anatomical-PT	− 0.365^*^	0.330^*^	− 0.303^*^	0.424^*^

*Anatomical-SS* anatomical sacral slope, *anatomical-PT* anatomical pelvic tilt, *PI* pelvic incidence, *SS* sacral slope, *APPA* anterior pelvic plane angle, *LCE* lateral center-edge angle, *ACE* anterior center-edge angle, *PCE* posterior center-edge angle, *AA* acetabular anteversion

*p < 0.01

With regard to validation, we obtained high intraobserver and interobserver reliability; the minimal ICCs for TK were 0.778 and 0.712 in intraobserver and interobserver ICCs, respectively (Table 4).

**Table 4:** Reliability of the measurement values

	Intraobserver reliability	Interobserver reliability
PI	0.848 (0.819–0.882)	0.753 (0.712–0.795)
SS	0.861 (0.836–0.895)	0.826 (0.789–0.862)
PT	0.844 (0.814–0.877)	0.727 (0.685–0.775)
Anatomical-SS	0.864 (0.836–0.897)	0.837 (0.809–0.872)
Anatomical-PT	0.832 (0.798–0.863)	0.722 (0.681–0.764)
TK	0.778 (0.738–0.822)	0.712 (0.671–0.756)
LL	0.839 (0.808–0.872)	0.742 (0.702–0.783)
APPA	0.852 (0.822–0.886)	0.768 (0.731–0.807)
LCE	0.961 (0.935–0.975)	0.939 (0.915–0.956)
ACE	0.975 (0.963–0.982)	0.950 (0.929–0.966)
PCE	0.958 (0.941–0.970)	0.919 (0.889–0.941)
AA	0.991 (0.978–0.998)	0.980 (0.967–0.988)

Data are presented as an interclass correlation coefficient (95% confidence interval)

*Anatomical-SS* anatomical sacral slope, *anatomical-PT* anatomical pelvic tilt, *LL* lumbar lordosis, *PI* pelvic incidence, *SS* sacral slope, *TK* thoracic kyphosis, *APPA* anterior pelvic plane angle, *ACE* anterior center-edge angle, *PCE* posterior center-edge angle, *AA* acetabular anteversion

With regard to the post hoc analysis, the power value was 0.803 in the correlation analysis.

## Discussion

In this study, we found that APPA had a stronger correlation with anatomical-PT than with bony morphological parameters of the acetabulum such as LCE, ACE, PCE, and AA, while there were significant correlations between APPA and both anatomical-PT and acetabular parameters. It was previously considered that DDH patients showed greater anterior tilt of the pelvis than individuals without DDH did. DDH patients have an anteriorly tilted pelvis to increase the bony coverage of the femoral head, according to Fukushima et al. [4]. All measurement values in Fukushima et al.’s study [4] were similar to those in our study: PT, 13.9° and 10.7°; PI, 55.1° and 54.2°; SS, 41.2° and 43.8°; LL, 54.5° and 55.4°; and LCE angle, 10.6° by plain radiograph and 13.9° by CT image, respectively. However, in this pervious study, only the LCE angle was compared with the anterior tilt of the pelvis. Further, Fukushima et al. reported that DDH patients had a lower LCE angle and higher SS and higher LL; subsequently, they speculated that DDH patients might tilt their pelvis anteriorly to increase the bony coverage of the femoral head, although they did not evaluate the correlation of them. Nevertheless, a similar correlation between the results of their study and those of our study may be obtained because the spino-pelvic parameters between both studies were similar.

In contrast, Fujii et al. [22] demonstrated a high AA and decreased anterior acetabular coverage by using CT images, similar to the current study. DDH is commonly known as one of the causes of secondary osteoarthritis of the hip [1]. The characteristics of the acetabulum of DDH patients are shallow and oblique. These abnormal deformities affect the elevated stress distribution in the narrow weight-bearing area in their hip joint, increase the shear stress at the acetabular edge, and lead to increased damage of the articular cartilage. Compared to the normal healthy women reported by Miyasaka et al. [23], women with DDH in our study had a lower LCE angle (31.6° versus [vs.] 13.9°), lower ACE angle (56.0° vs. 40.6°), lower PCE angle (102.9° vs. 99.8°), and higher anteversion (23.6° vs. 29.6°). Hence, higher anterior tilt of the pelvis or higher APPA may be more associated with anatomical-PT, a spino-pelvic morphological parameter, than with lower bony coverage of the femoral head by acetabulum. Therefore, anterior tilt of the pelvis may be more dependent on spino-pelvic morphological parameters such as anatomical-PT than on increased bony coverage of the femoral head. We speculated that PT in DDH patients was also adjusted to a constant value, maybe approximately 10°, which is the average value in this study to tilt the pelvis anteriorly; thus, patients with higher anatomical-PT may tilt their pelvis more anteriorly. Subsequently, there was a significant correlation between anatomical-PT and APPA.

This study had several limitations. First, the number of participants in the study was small. Second, the subjects were only female patients. It has been reported that the prevalence of DDH has female predominance with a 9:1 female to male ratio [24]. Further, there were only 20 male patients who had undergone curved periacetabular osteotomy during the last 10 years in author’s institution. Accordingly, similar examination should be conducted in male subjects in the future. Third, the participants of this study did not have severe osteoarthritis of the hip. Although it is important to evaluate these measurements in patients with severe arthritic changes, it may be difficult to accurately evaluate the angles in patients with aspherical and/or squamous femoral heads.

This study also has a strong point. Although they were all women, the participants in this study had DDH bilaterally that was evaluated as less than 2 according to Crowe’s classification. A similar previous study included patients with bilateral congenital hip dislocation [25] or only evaluated unilateral hip disease without evaluating the condition of the contralateral side [4]. Therefore, the patient bias in our study seems to be comparatively less than that in other previous studies. Moreover, this study was the first report to investigate the correlation between functional parameters of the pelvis and spine and morphological parameters of the pelvis and acetabulum besides PI. Further examination is required to determine whether APPA is dependent on anatomical-PT after pelvis osteotomy or THA. If the correlation between postoperative APPA and anatomical-PT after pelvic osteotomy or THA was confirmed, the surgeon may be able to plan the position of the fragment in pelvic osteotomy or cup positioning separately from cup inclination and anteversion considering postoperative anatomical-PT.

## Conclusions

Tilt of the pelvis may be more dependent on anatomical-PT, a morphological parameter of the pelvis, than on the bony coverage of the acetabulum such as LCE, ACE, PCE, and AA. This study is the first to investigate the correlation between functional parameters of the pelvis and spine and morphological parameters of the pelvis and acetabulum besides PI.

## Data Availability

Not applicable.

## Abbreviations

3D: Three-dimensional
AA: Acetabular anteversion
ACE: Anterior center-edge angle
Anatomical-PT: Anatomical pelvic tilt
Anatomical-SS: Anatomical sacral slope
APP: Anterior pelvic plane
APPA: Anterior pelvic plane angle
ASISs: Anterior superior iliac spines
CT: Computed tomography
DDH: Developmental dysplasia of the hip
ICCs: Intraclass correlation coefficients
LCE: Lateral center-edge angle
LL: Lumbar lordosis
PCE: Posterior center-edge angle
PI: Pelvic incidence
PT: Pelvic tilt
SS: Sacral slope
vs.: Versus
